# Supplementary figures and images for: PINNing cerebral blood flow: analysis of perfusion MRI in infants using physics-informed neural networks
Source: Front Netw Physiol. 2025 Feb 14;5:1488349. doi: 10.3389/fnetp.2025.1488349 (PMC11868054; doi:10.3389/fnetp.2025.1488349)

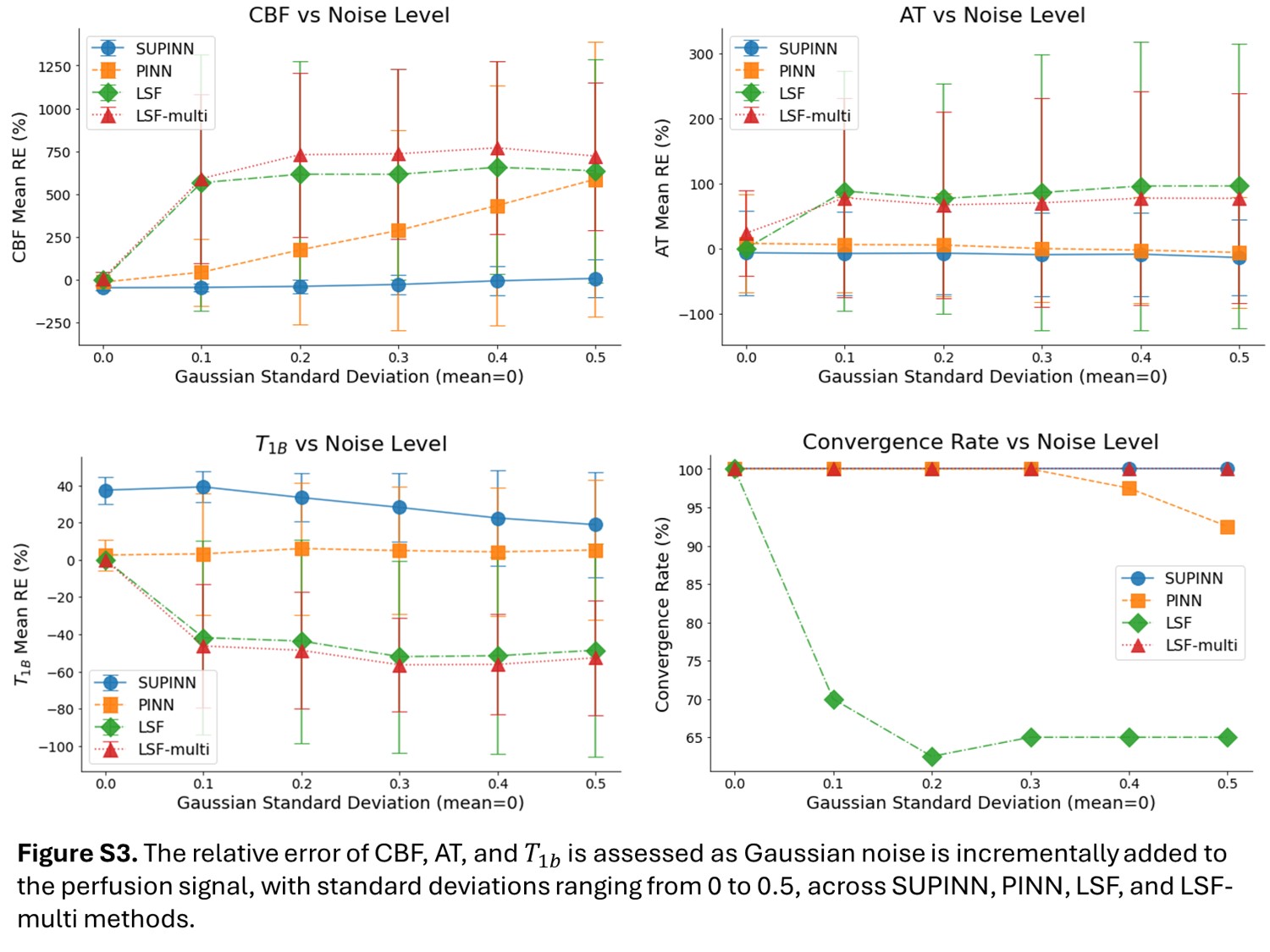

Supplement: Supplementary file 1 [file Image3.jpeg]

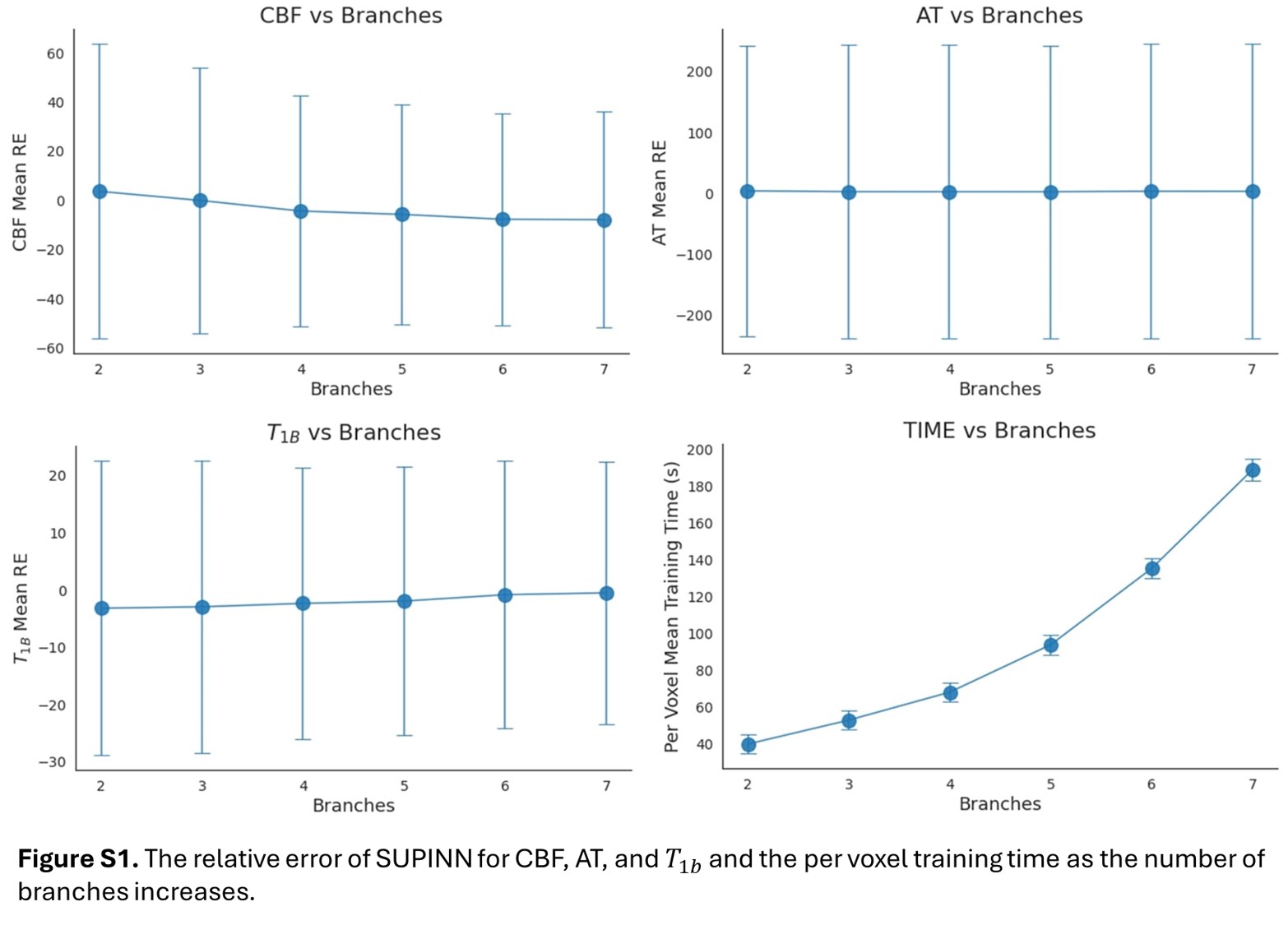

Supplement: Supplementary file 3 [file Image1.jpeg]

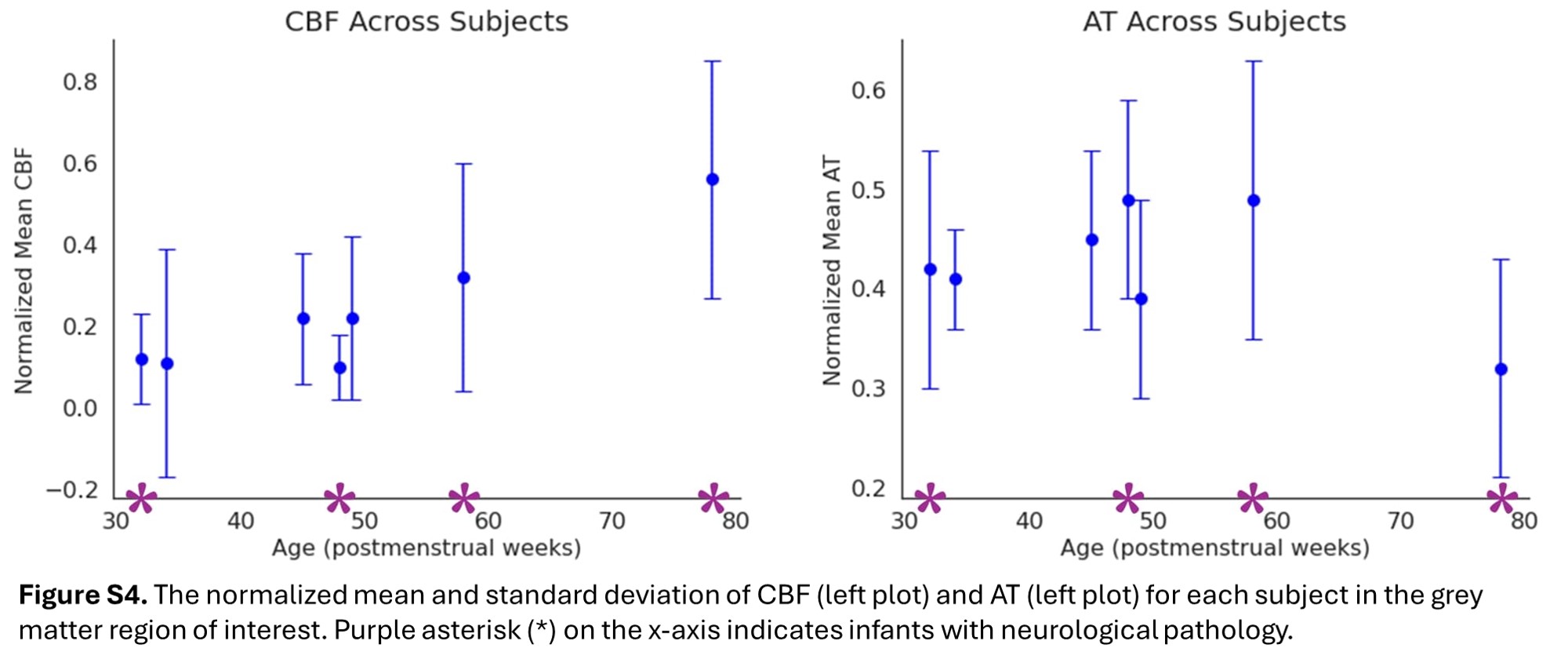

Supplement: Supplementary file 4 [file Image4.jpeg]

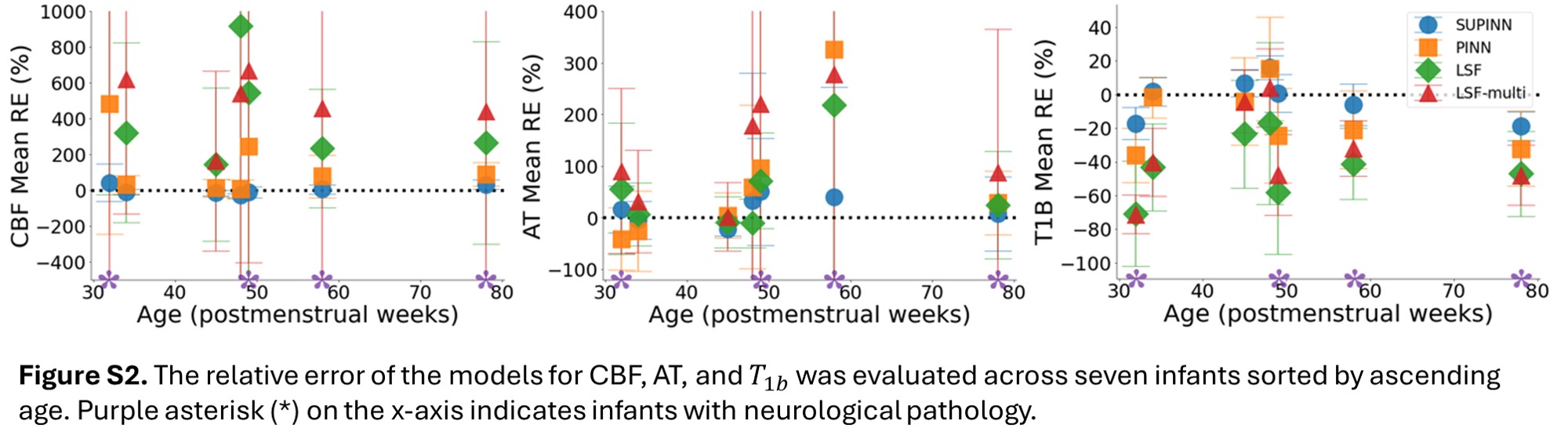

Supplement: Supplementary file 5 [file Image2.jpeg]
