# Supplementary material for: PINNing cerebral blood flow: analysis of perfusion MRI in infants using physics-informed neural networks
Source: Front Netw Physiol. 2025 Feb 14;5:1488349. doi: 10.3389/fnetp.2025.1488349 (PMC11868054; doi:10.3389/fnetp.2025.1488349)
Supplement: Supplementary file 2 [file Table1.docx]

Table S1. Definitions for a smooth hyperbolic 3-branch ODE.

| **Function** | **Phase** | **Definition** |
| --- | --- | --- |
| Hyperbolic Smoothing | Arrival | $h_{2}=(0.5+0.5\tanh\left( \frac{t-AT}{0.1} \right))\times(0.5-0.5\tanh\left( \frac{t-AT-\tau}{0.1} \right))$ |
| Hyperbolic Smoothing | Diffusion | $h_{3}=0.5+0.5\tanh\left( \frac{t-AT-\tau}{0.1} \right)$ |
| ODE | Pre-Arrival | $\frac{{ds}_{1}}{dt}=0$ |
| ODE | Arrival | $\frac{{ds}_{2}}{dt}=CBF\times e^{\frac{-t}{T_{1b}}}\times\left( 1-\frac{t-AT}{T_{1b}} \right)$ |
| ODE | Diffusion | $\frac{{ds}_{3}}{dt}=-CBF\times e^{\frac{-t}{T_{1b}}}\times\frac{\tau}{T_{1b}}$ |
| ODE | Continuous | $\frac{ds}{dt}=\frac{{ds}_{1}}{dt}+\frac{{ds}_{2}}{dt}\times h_{2}+\frac{{ds}_{3}}{dt}\times h_{3}$ |
