# Supplementary material for: PINNing cerebral blood flow: analysis of perfusion MRI in infants using physics-informed neural networks
Source: Front Netw Physiol. 2025 Feb 14;5:1488349. doi: 10.3389/fnetp.2025.1488349 (PMC11868054; doi:10.3389/fnetp.2025.1488349)
Supplement: Supplementary file 6 [file Table2.docx]

Table S2. Summary of hierarchical optimization parameters.

| **Level** | **Optimizer** | **Learning Rate** | **Decay Rate/per steps** | **Iterations** |
| --- | --- | --- | --- | --- |
| Macro | Adam | 0.001 | 0.2/500 | 5,000 |
| Meso | Adam | 0.0001 | 0.15/2,000 | 20,000 |
| Micro | L-BFGS | N/A | N/A | 100 |
